# Evaluating generative AI for qualitative data extraction in community-based fisheries management literature

**DOI:** 10.1186/s13750-025-00362-9

**Published:** 2025-06-02

**Authors:** S. Spillias, K. M. Ollerhead, M. Andreotta, R. Annand-Jones, F. Boschetti, J. Duggan, D. B. Karcher, C. Paris, R. J. Shellock, R. Trebilco

**Affiliations:** 1 CSIRO Environment, Hobart, TAS Australia; 2 CSIRO Environment, Perth, WA Australia; 3 Centre for Marine Socioecology, University of Tasmania, Hobart, TAS Australia; 4 Fenner School of Environment and Society, Australian National University, Canberra, ACT Australia; 5 Australian National Centre for the Public Awareness of Science, Australian National University, Canberra, ACT Australia; 6 Centre for Sustainable Development Reform, Faculty of Law and Justice, University of New South Wales Sydney, Sydney, NSW Australia; 7 CSIRO Data61, Sydney, NSW Australia

**Keywords:** Artificial Intelligence, Systematic review, Large language models, Scientific publication, Natural-language processing, Future of science

## Abstract

Uptake of AI tools in knowledge production processes is rapidly growing. In this pilot study, we explore the ability of generative AI tools to reliably extract qualitative data from a limited sample of peer-reviewed documents in the context of community-based fisheries management (CBFM) literature. Specifically, we evaluate the capacity of multiple AI tools to analyse 33 CBFM papers and extract relevant information for a systematic literature review, comparing the results to those of human reviewers. We address how well AI tools can discern the presence of relevant contextual data, whether the outputs of AI tools are comparable to human extractions, and whether the difficulty of question influences the performance of the extraction. While the AI tools we tested (GPT4-Turbo and Elicit) were not reliable in discerning the presence or absence of contextual data, at least one of the AI tools consistently returned responses that were on par with human reviewers. These results highlight the potential utility of AI tools in the extraction phase of evidence synthesis for supporting human-led reviews, while underscoring the ongoing need for human oversight. This exploratory investigation provides initial insights into the current capabilities and limitations of AI in qualitative data extraction within the specific domain of CBFM, laying groundwork for future, more comprehensive evaluations across diverse fields and larger datasets.

**Supplementary Information:**

The online version contains supplementary material available at 10.1186/s13750-025-00362-9.

## Introduction

Evidence-informed decision-making entails identifying, appraising and mobilising the best available evidence for the design and implementation of policy, programs and interventions [1]. It is increasingly expected that policy, planning and management decisions are made using the best available evidence, and this is essential for navigating the contemporary environmental challenges that threaten human well-being and prosperity [2]. However, there are various barriers to the use of environmental evidence in decision-making. The most common barriers relate to: (i) the nature of the evidence itself (i.e., its accessibility, relevance, applicability and quality), (ii) the capacity and resources of decision-makers to identify, access and read the evidence, (iii) the relationships and links between scientists and decision-makers, and (iv) the structure of decision-making processes themselves, which may not provide adequate space or mechanisms for evidence integration [3, 4].

Large Language Models (LLMs) and generative AI technologies represent a significant advancement in automated text analysis and understanding. LLMs are neural networks trained on vast amounts of writing to perform Natural Language Processing tasks, such as generating human-like text, while generative AI encompasses a broader family of AI systems capable of creating new content based on training data [5]. These technologies have shown remarkable capabilities in tasks ranging from text summarization to complex reasoning, with their abilities expanding rapidly through architectural improvements and enhanced training approaches [6]. Understanding their potential and limitations is crucial as they become increasingly integrated into academic research workflows.

Evidence syntheses, which use robust and transparent methods to combine information from multiple studies [7], are a key tool for addressing these challenges. While these methods provide rigorous frameworks for evaluating and summarizing literature [8, 9], they are increasingly challenging to implement as the volume of academic literature grows. In 2022 alone, an estimated 5.14 million academic articles were published [10], with this growth likely to accelerate due to the adoption of generative AI tools in academic writing [11]. The resource-intensive nature of evidence syntheses, combined with inherent reviewer subjectivity [3], creates a pressing need for more efficient and systematic approaches to literature analysis.

One potential solution to this problem is the use of Large Language Models (LLMs) and other generative AI technologies to identify relevant articles and extract the desired information [6]. The capability of these technologies is accelerating extraordinarily rapidly, with new advancements being released constantly [5]. Previous research has shown that LLMs can be beneficial for systematic evidence syntheses, especially during the initial paper screening process [12–17]. Historically, a range of natural language processing algorithms have been used to extract entities or metadata from either abstracts or full-texts in the literature [18–20]. Our previous research has shown that general-use Large Language Models like ChatGPT, which can be easily applied to novel domains without extensive training or data curation by the user, can improve the reliability of screening for literature [15]. There is mounting evidence that LLMs and other generative AI tools can be used to reliably extract structured information from scientific text [17, 20–22] and specifically ecological text [23–26]. While recent ecological research has shown that LLMs can achieve high accuracy (>90%) when extracting discrete and categorical data, they still struggle with certain types of quantitative information extraction [24]. Moreover, these studies have primarily focused on well-defined, structured information extraction tasks where the target data is explicitly stated in the text. The ability of LLMs to answer nuanced questions about context requiring synthesis and interpretation of qualitative information across full scientific papers remains largely unexplored, particularly in domains like environmental management where information may be dispersed and where sophisticated understanding of social-ecological contexts is required.

**Fig. 1:**
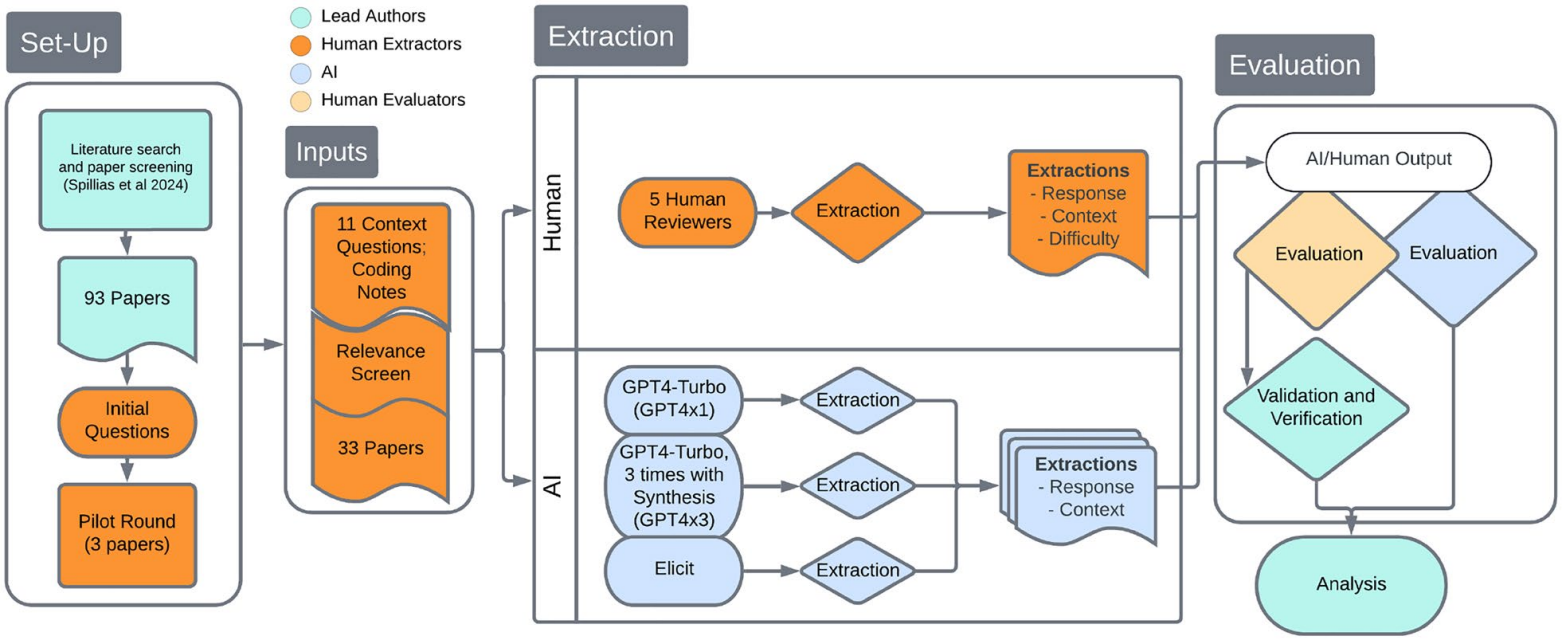
Conceptual diagram of the methods. The study employed a dual-extraction and evaluation method, with human reviewers and LLMs analysing literature on community-based fisheries management (CBFM) and a separate team of human and LLM evaluators assessing the quality of the LLM outputs

To address this gap, we conducted a pilot study, focusing specifically on CBFM literature. Community-based fisheries management (CBFM) literature presents a particularly interesting test case for evaluating LLMs’ capabilities in qualitative data extraction. Unlike simpler extraction tasks, CBFM literature often contains nuanced discussions of governance structures, power dynamics, and complex social-ecological relationships that require sophisticated contextual understanding to interpret correctly. These papers frequently describe qualitative aspects of management systems, such as access rights, community engagement, and traditional knowledge integration—topics that may challenge LLMs’ ability to accurately extract and synthesize information. This exploratory investigation evaluated the capacity of three LLM implementations to extract relevant contextual information from a small sample of 33 peer-reviewed articles and compared it to that of trained human experts. We focused on a case study of benefits and barriers of CBFM in Pacific island states, building upon our previous research that used this case study to evaluate the ability of ChatGPT to search peer-reviewed literature and screen papers for relevance [15]. For this example research topic, we posed 11 contextual questions to ask of the set of peer-reviewed papers identified in Spillias et al., [15], aimed at generating a qualitative data set that could be used to synthesise the current state of evidence-based knowledge in this field. We used three LLM implementations: two based on GPT4-Turbo and one using Elicit.com, which employs LLMs within a specialized architecture for academic literature extraction [27]. One team of human experts (extractors) extracted information from the 33 papers [see Additional file 1 for included papers], and then a separate team of human experts evaluated the quality of LLM responses across three criteria (evaluators; see Methods). With this procedure (Fig. 1), we aimed to answer the following research questions:

1. How well can LLMs discern the presence or absence of relevant contextual data in CBFM literature?
2. How does the quality of LLM extraction outputs compare to those done by human researchers in the context of CBFM?
3. Does the difficulty or type of question influence the performance of the extraction in CBFM-related queries?

While this pilot study focuses specifically on CBFM literature, it provides valuable initial insights into the potential and limitations of using LLMs for qualitative data extraction in systematic reviews. The findings from this focused exploration can inform future, more comprehensive studies across diverse fields and contribute to the ongoing development of LLM-assisted evidence synthesis methodologies.

## Methods

### Overview

This pilot study undertook a parallel data extraction process with a team of human reviewers and a set of LLM implementations, followed by an evaluation of the LLM responses using an independent evaluation team. Two teams were assembled and each given a set of relevant literature on community-based fisheries management (CBFM) for analysis. The first team consisted of human extractors (KO, JD, DK, RS, RAJ) with experience in various forms of evidence synthesis (e.g., [28, 29]), who undertook a traditional evidence extraction from a set of peer-reviewed literature. The other team (SS, FB, MA, RT) with modelling expertise, employed three LLM implementations to produce outputs in accordance with the methods that would be followed by the first team. Both the human reviewers and LLM models were asked to answer a series of qualitative questions about each of the CBFM-related papers. The LLM-enabled team then compared the human responses to the LLM model responses and ranked the level of similarity.

### Topic selection

Given the expertise of the human reviewers and the previous screening research [15], this case study focused on investigating and annotating data from the existing literature on community-based fisheries management (CBFM). CBFM is an approach to fisheries management where local coastal communities and fishers are responsible for managing their coastal region and resources in an effort to ensure their sustainable use [30]. We chose this specific topic for several reasons:

1. It provided a well-defined and coherent body of literature for our pilot study.
2. The variability and diversity of terminology and language used in CBFM research offered a robust test of the LLMs’ capabilities to extract and synthesise relevant information.
3. It allowed us to build upon our team’s existing expertise and previous work in this area.
4. The topic provided the opportunity to elicit a range of data types and complexities to test LLM extraction capabilities.

While this focused approach limits the generalizability of our findings, it allows for an in-depth exploration of LLM performance in a specific domain, which can inform future, broader studies.

### Human extraction procedure

The human reviewers developed and refined the extraction questions through an iterative process using three randomly chosen papers as a training set. Both human reviewers and LLMs analyzed these papers, with the human responses compiled and compared by one reviewer (KO) to identify areas of inconsistency. The human team then met to discuss divergent interpretations, refining question phrasing and adding coding notes until consistent interpretation was achieved among human reviewers. While LLM responses helped identify unclear or ambiguous phrasings, agreement with AI outputs was not sought during this development phase. This process led to our final set of questions with accompanying coding notes that provided specific examples and clarified potential ambiguities.

This refinement process led to our final set of eleven questions, each accompanied by detailed coding notes that provided specific examples and clarified potential ambiguities (Table 1). The questions were deliberately designed to cover a range of information types and response formats. Most questions required open-text responses allowing for detailed qualitative analysis, with one, the question about stakeholder groups involved in management, included predefined categories (Community-members, Researchers, Practitioners, Government, NGOs, Other). Even seemingly straightforward questions like country location were designed as open-text responses rather than strict classifications, as our pilot testing revealed that many papers discussed multiple countries or regional contexts that would not fit neatly into predefined categories. This variety in question types and response formats allowed us to test the LLMs’ capabilities across different types of information extraction tasks, from simple factual identification to complex qualitative synthesis.

**Table 1:** Contextual questions and coding notes posed to the human and LLM extractors

Question	Coding notes
Which country was the study conducted in?	If multiple countries are part of a single case study, or if there are multiple case studies—code them separately at the country level
Provide some background as to the drivers and/or motivators of community-based fisheries management.	Why is the CBFM in place/being used? Separate to the benefits of the CBFM—could be because there is strong existing community ownership, could be because all other approaches have failed, could be because of an inherent mistrust of government?
What management mechanisms are used?	Tangible mechanisms being used to manage the fishery—could be physical limitations like gear limits, size limits or timing limits.
Which groups of people are involved in the management as part of the CBFM case-studies? Choices: Community-members, Researchers, Practitioners, Government, NGOs, Other	Involved directly in the management of the fishery, NOT in the conducting of the study (collecting data for the study)
What benefits of Community-Based Fisheries Management are reported in this case study?	Physical or social-ecological benefits—could include things like increased fish counts or larger fish size, or improved social outcomes for communities
What are the indicators of success of CBFM?	What are the data sources for measuring success—community perception, fish size etc.
How was the data on benefits collected?	What methods were adopted—catch and release programs, qualitative survey etc.
What are the reported barriers to success of Community-Based Fisheries Management?	Focus on things that are reported around the case study in question—NOT general examples. What was hindering the success of the CBFM—e.g. poor community buy in, poaching etc.
Guidelines for future implementation of CBFM?	This can be more general. In light of the study, what are the take home messages for future CBFM projects—NOT future research directions, or things to consider for future studies
How does the community monitor the system they are managing?	Within the case study, what are the community groups you have already identified doing to monitor the fishery—are they conducting fish surveys, monitoring community catches etc
How does the community make decisions?	How do they make decisions around the management of the fishery—eg. all decisions are passed on by the matai, or individual villages make decisions on anything that happens from their shoreline.

After the pilot round, 93 papers found in Spillias et al. [15] were randomly distributed equally among the five human reviewers. When reading the full-text paper, the reviewers could exclude the paper from further review if they thought that it did not fit the research question and/or if the extraction questions could not be answered based on the content. The reviewers excluded 60 papers this way, leaving 33 papers for the full analysis.

It’s important to note the constraints that led to this sample size. The initial pool of 93 papers was determined by the availability of relevant CBFM literature identified in our previous work [15]. The exclusion of 60 papers was necessary to ensure the quality and relevance of our dataset, as these papers either did not align with our specific research questions or lacked sufficient information to answer our extraction questions. This rigorous selection process, while reducing our sample size, allowed us to focus on papers that provided rich, relevant data for our analysis.

The resulting sample size of 33 papers, while relatively small, was deemed sufficient for this pilot study. It allowed for a detailed, in-depth analysis of LLM performance on a specific set of CBFM literature, which aligns with our goal of conducting an exploratory investigation. However, we acknowledge that this limited sample size impacts the statistical power of our analysis and the broader applicability of our results, a limitation we discuss further in our conclusions.

For each question and paper, the reviewers gave a short answer to the question (Response) and identified one or more passages that supported the answer (Context). If no answer was possible because the information was not in the paper, no answer was given. Because we were interested in identifying potential drivers of quality in LLM extracted results, the human reviewers also recorded their perceived difficulty in answering the extraction question for each paper as either Easy, Medium, or Hard. These difficulty ratings were based on the reviewers’ expertise in evidence synthesis and reflected whether answers were plainly stated in the text or required more complex interpretation. We did not ask reviewers to provide detailed explanations of their ratings, as we wanted to capture their overall assessment as experienced practitioners. This perceived difficulty rating was then used as one dimension to test whether the complexity of information extraction, from a human perspective, influenced the quality of AI responses.

### LLM extraction procedure

We selected three distinct LLM implementations to evaluate different approaches to information extraction, each chosen for specific methodological reasons. First, we used a single call to GPT4-Turbo (GPT4x1) as a baseline implementation, representing the simplest possible approach that a researcher might take. Second, we implemented a more sophisticated approach using three calls to GPT4-Turbo followed by synthesis (GPT4x3), testing whether multiple independent extractions could improve reliability, as found in [15]. Finally, we employed Elicit’s specialised academic extraction system to evaluate how a purpose-built tool might compare to general-purpose LLMs. This selection of implementations allowed us to compare both simple and complex approaches, as well as general versus specialised tools. The scripts used to access GPT4-Turbo via the API are available online at (https://github.com/s-spillias/AI_Extraction).

We accessed GPT4-Turbo using the Microsoft Azure API in late January 2024. The prompting strategies differed significantly between implementations. For GPT4x1, we used a straightforward prompt structure that mirrored the human procedure, explicitly requesting both a Response and supporting Context, with clear instructions to indicate when no relevant information was found. The prompt was carefully designed to encourage precise, focused responses while maintaining consistency with human extraction guidelines. For GPT4x3, we used the same base prompt but implemented it three times independently, followed by a synthesis step that combined the responses while preserving all identified context passages. This approach was designed to leverage the non-deterministic nature of LLM responses to potentially capture different aspects of the information.

Each implementation received a cleaned version of the paper text with metadata and backmatter removed to ensure consistent input quality. The Context passages in GPT4x3 were automatically concatenated rather than synthesised to preserve the direct connection to source material.

**Fig. 2:**
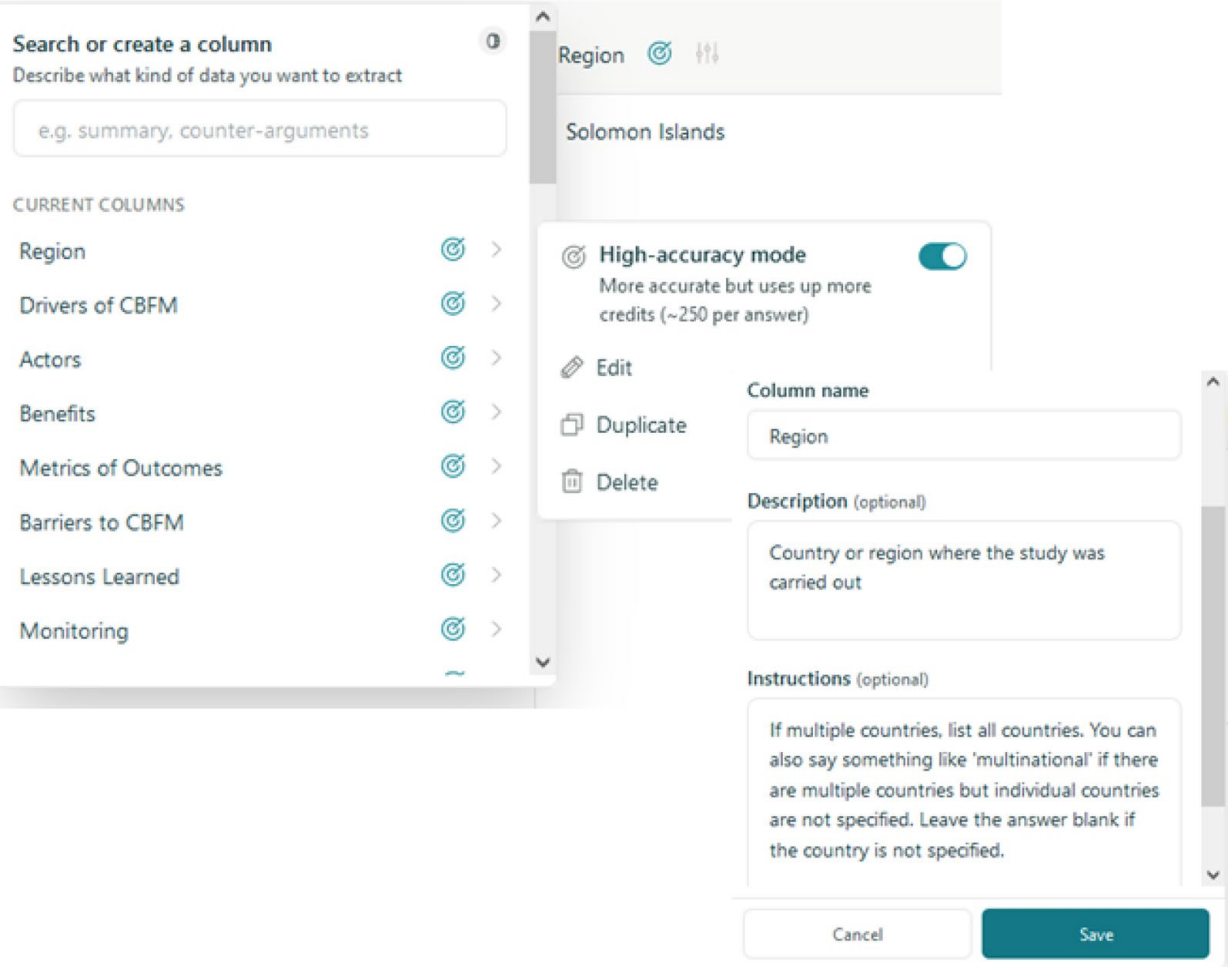
Screenshot of the Elicit interface showing the data extraction setup. The interface allows users to upload PDFs and configure extraction parameters including column names, descriptions, and instructions. The ’High-accuracy Mode’ setting enables additional verification steps in the extraction pipeline

To provide a more sophisticated approach to information extraction, we also employed Elicit, which represents a purpose-built architecture specifically designed for academic literature extraction [27]. Unlike our relatively straightforward GPT4 implementations, Elicit employs a complex system of LLMs combined with specialised processing techniques aimed at extracting relevant and truthful information from scientific papers. We uploaded the papers as PDFs to Elicit’s online portal on January 29th 2024, providing a short column name for each question and putting the unaltered text of the question and explanation into the ‘Description’ and ‘Instructions’ fields, respectively (Fig. 2). We also enabled the feature ’High-accuracy Mode’ for all columns, which activates additional verification steps in Elicit’s extraction pipeline. Elicit automatically returns both ’Supporting’ and ’Reasoning’ passages from the text to support the response provided, which we used as ’Context’ for our evaluation.

For the context strings returned by the LLMs, we verified their presence in the articles (i.e., that the models had not ’hallucinated’), by string matching the context returned by the LLMs with the full-text of the article. We identified 29 instances where the Context returned by the LLMs did not match any strings in the full-text. We manually investigated each one and confirmed that, with the exception of one unique Context string, all of the Context strings were present in their respective articles, indicating essentially near-zero rates of hallucination.

### Evaluation procedure

Following the human extraction process, and working independently from the extraction team, an evaluation team (SS, MA, RT, FB) developed a procedure for evaluating the quality of the LLM outputs in comparison to the human extractions. Initially, the possibility of a fully blind process was considered, where the source of the extractions (human or LLM) would not be revealed to the evaluators. However, this approach was ultimately rejected because we were concerned that if the evaluation team did not know the source of the extractions, then they would lack a proper baseline for judging the quality of the responses. Establishing a gold standard, is common practice in natural-language processing for evaluating the quality of algorithmic outputs [31], therefore we decided this would be an appropriate initial benchmark. Consequently, while the evaluators were aware of which extractions were LLM-generated and which were human-generated, they were not informed about which specific LLM model produced each extraction. This partial blinding was intended to reduce bias in the evaluation process while still providing a reference point for quality assessment. The resulting procedure is reflective of evaluation processes developed by other groups [32].

**Fig. 3:**
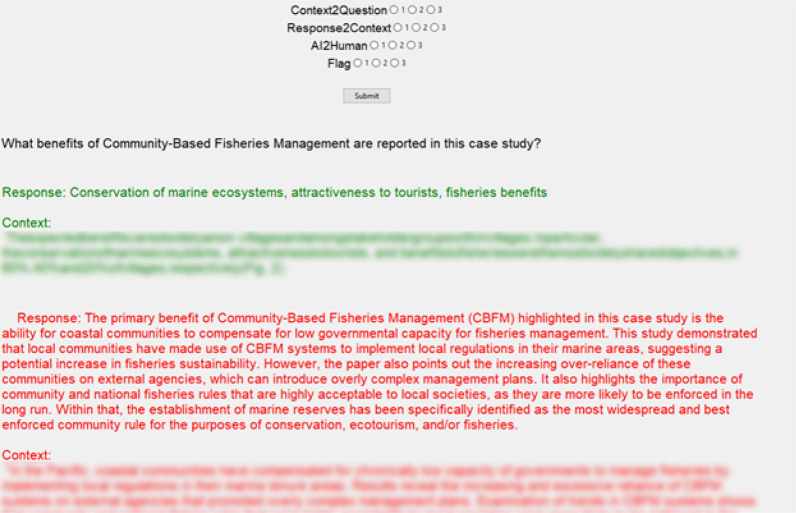
Screenshot of the custom evaluation interface used by human evaluators. The interface displays the question being evaluated, the human and LLM responses with their supporting context, and allows evaluators to rate the quality of the LLM output across three criteria using a three-point scale

To assess the quality of the LLM-generated responses, the evaluation team established three criteria: (i) whether the Context provided by the LLM was appropriate evidence give the question that had been posed; (ii) whether the Response was an appropriate synthesis of the Context in response to the extraction question; and (iii) how the LLM output compared to the human output, with the human extractions serving as a ’Gold Standard’. A three-point scale- −1 for Poor, 0 for Fair, and 1 for Good-was employed by the evaluation team for grading purposes. A custom program was designed in Python to facilitate this evaluation process (Figure 3). We also performed the same evaluation procedure using GPT4-Turbo as an evaluator to provide further support for the assessments of the human evaluators. This was done through the API and was repeated five times for each question-paper pair.

During the initial assessment, the evaluation team found that some responses from the LLM required additional context, beyond what the LLM had reported, from the paper to evaluate. Therefore a Flag criterion was added to the custom program for further follow-up to investigate the possibility that LLM responses had provided more detailed and/or accurate information than the Gold Standard human extraction.

### Follow-up verification

We performed two manual follow-up verification checks to ensure that the LLMs were returning valid responses from the articles. First, for every LLM output, we verified that the Context returned by the model was indeed present in the full text article. We did this using an automated script that involved a three-step verification process. This process was designed to account for potential discrepancies due to OCR errors, formatting changes, and minor variations in the text. The first step in the process involved normalising the context string extracted by the LLM and the corresponding passage from the full-text article. Normalisation entailed removing all punctuation, converting all characters to lowercase, and replacing newline characters with spaces. This step reduces the variability between strings caused by differences in formatting and case sensitivity. The normalised strings were then compared for similarity. We utilised the fuzz.partial_ratio function from the fuzzywuzzy library to calculate the degree of similarity between the LLM-generated context and the text from the full article. If the similarity score exceeded a predefined threshold of 90%, the strings were considered similar enough to be a match. This step allows for a degree of tolerance in the match, accommodating minor differences in wording or spelling that might occur. In addition to the string similarity check, we also performed a cosine similarity calculation using the CountVectorizer from the scikit-learn library [33]. The two normalised strings were transformed into a document-term matrix, representing the frequency of terms within each string. The cosine similarity between these two term frequency vectors was then calculated. A cosine similarity score greater than 0.5 was considered indicative of a significant match between the strings. The context string was considered valid if it was contained within the full-text passage, exhibited a high degree of similarity with the passage, or had a cosine similarity score indicating a strong match. By employing these methods, we were able to robustly verify the LLM-generated context against the source articles, ensuring that the models did not ’hallucinate’ or fabricate information not present in the original texts.

Second, the evaluation team had the opportunity to ’flag’ question/output pairs that they felt warranted additional investigation. For each of these flagged data points, two authors (SS, KO) manually investigated the LLM response and assessed whether it was faithfully reporting information from the paper.

### Statistical analysis

We calculated the inter-rater reliability by using Cohen’s kappa statistic to evaluate the agreement between the presence/absence of contextual data between the human reviewers and the LLM implementations.

We created confusion matrices to compare the frequencies at which each LLM implementation and human extractors returned similar responses by either providing a response (data) or not (no data) to a given question from each paper. This was calculated separately for each LLM implementation (Elicit, GPT4x1, and GPT4x3) and independently for each response value. All values in each confusion matrix are reported as a portion of the total of 1. These matrices have four quadrants and shows how frequently: the human did not provide a response but the LLM did (quadrant 1, top right, false positive), the human and the LLM both provided a response (quadrant 2, top left, true positive), the human provided a response but the LLM did not (quadrant 3, bottom left, false negative), and neither the human or LLM provided a response (quadrant 4, bottom right, true negative).

Several criteria were measured throughout this study including how well the LLM pulled the relevant context from the paper for the specific question (Context to Question), how well the LLM responded to the relevant context (Response to Context), and how the LLM response compared to the human response (LLM Response to Human Response).

To analyse all the results, we performed statistical analyses using R software [34]. Significance was determined using an $\alpha$-critical level of 0.05 for all tests. A two-way t-test was done to allow for comparison between assessed values for each LLM implementation and each criteria against the ‘fair’ extraction score of 0. In subsequent analyses, we employed a linear mixed-modelling approach (lmer function from lme4 package [35]) to assess the impacts of different factors (LLM implementations, difficulty, and questions) on overall assessed values. Where applicable, evaluator, extractor, and paper were included as random effects. An analysis of variance (ANOVA) test was performed to assess the overall significance of each variable. When required, pairwise comparisons were then performed on the estimated marginal means (emmeans and pairs functions from emmeans package [36]) and Tukey’s method was applied to determine significance of comparison.

To examine the overall results, we created a linear mixed-effects model showing the effect of each LLM implementation and criteria on assessed quality. This model included the effects of the LLM implementations and the interaction of criteria as fixed effects, with evaluator and paper as random variables. Additionally, we constructed individual models to assess the effect of the LLM implementation on assessed quality for each criterion independently.

We then investigated the impacts of the questions on the assessed quality of the LLM responses using a linear mixed-effects model with the question being the fixed effect and evaluator and paper as random effects.

To evaluate the effect of difficulty, as ranked by the human extraction team, on the assessed quality, we used a linear mixed-effects model including the interaction between difficulty and LLM as the fixed effect, with paper and extractor as random effects. The model investigates the relationship between the assessed quality of each question-paper pair and the interaction between the ranked difficulty of the data point and the type of LLM implementation.

Finally, a linear model was also created to ensure that the extractor did not have an effect on the assessed quality.

### Response length analysis

To analyze differences in verbosity between human and LLM responses, we developed a Python script to process the extraction files and calculate word counts for each response. The script sanitised the text by removing excess whitespace and standardizing formatting, then counted words by splitting on spaces. We excluded responses marked as ’NO CONTEXT’ or ’NO DATA’ and those with fewer than 40 characters for human responses or 45 characters for LLM responses, following the same filtering criteria used in our quality assessment.

We performed one-way ANOVA followed by pairwise t-tests with Bonferroni correction ($\alpha$ = 0.05/6 for six pairwise comparisons) to assess statistical significance of differences in response lengths between human extractors and each LLM implementation. To examine whether response length influenced extraction quality, we analyzed the correlation between response length and quality scores using Pearson’s correlation coefficient. We also segmented responses into length ranges (0–100, 101–200, 201–300, 301–400, and 401+ words) to examine how mean quality scores varied across different levels of verbosity. Quality scores were derived from the third component of our evaluation criteria, which assessed how well the LLM responses compared to human responses on a scale from 1 (poor) to 3 (good).

## Results

### Discerning the existence of contextual data

**Fig. 4:**
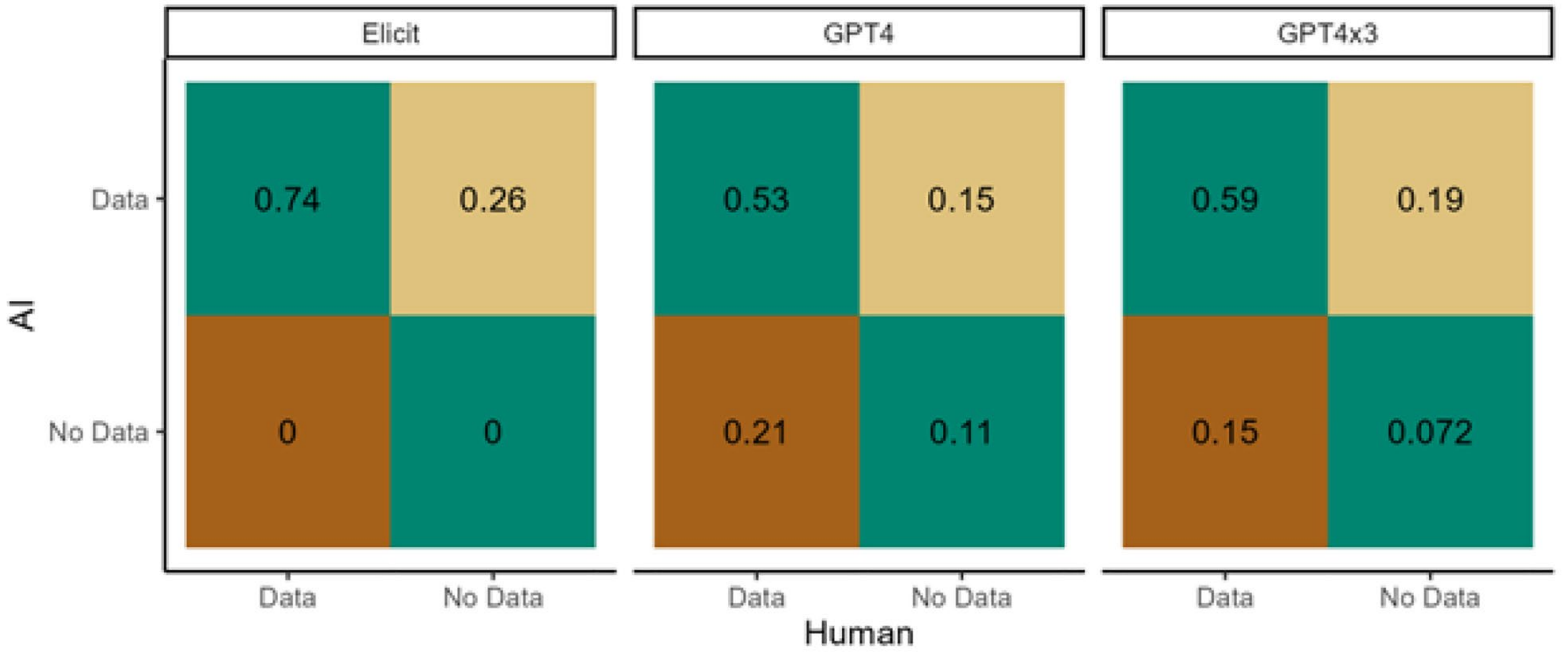
Agreement between LLMs and human reviewers on the presence or absence of contextual data in 363 paper-question pairs. The green quadrants show agreement rates: the top-left quadrant indicates the percentage of pairs where both LLMs and human reviewers agree that contextual data is present, while the bottom-right quadrant shows agreement on the absence of data. The top-right quadrant highlights false positives, where LLMs identified data that human reviewers did not, and the bottom-left quadrant indicates false negatives, where human reviewers identified data that LLMs missed. False negatives are particularly critical as they result in the omission of potentially relevant information, whereas false positives can be subsequently filtered out during evidence synthesis

The human reviewers analyzed 33 papers [see Additional file 1] according to 11 extraction questions and recorded the presence of contextual data for 74% of the 363 paper-question pairs (e.g., for a given study, a response was recorded for a question such as ‘Which country was the study conducted in?’) (Fig. 4). For the other 26% paper-question pairs, the reviewers determined that there was an absence of relevant data or context for the paper-question pair (The outputs from the human and LLM extractions are available at (https://github.com/s-spillias/AI_Extraction)).

The presence/absence results for the three LLM implementations (GPT4x1, GPT4x3, Elicit) had very low agreement (inter-rater reliability; Cohen’s kappa) with the human reviewers’ extraction (kappa < 0.10). Elicit’s agreement was especially low (kappa < 0.0), because it returned responses for all but one paper-question pair (> 99% presence), which resulted in a high rate of false positives (27%), but a near-zero rate of false negatives. The single run of GPT4-Turbo (GPT4x1) agreed with the presence/absence determination of the human reviewers in 63% of the questions and had a false positive rate of 15% and a false negative rate of 21%. The triple run of GPT4-Turbo (GPT4x3) performed slightly better, with agreement with 66% of the human reviewers’ paper-question pairs. Compared to GPT4x1, GPT4x3 also reduced the number of false negatives from 21 to 15%, but increased the false positive rate from 15 to 19%.

### Quality of LLM extractions

**Fig. 5:**
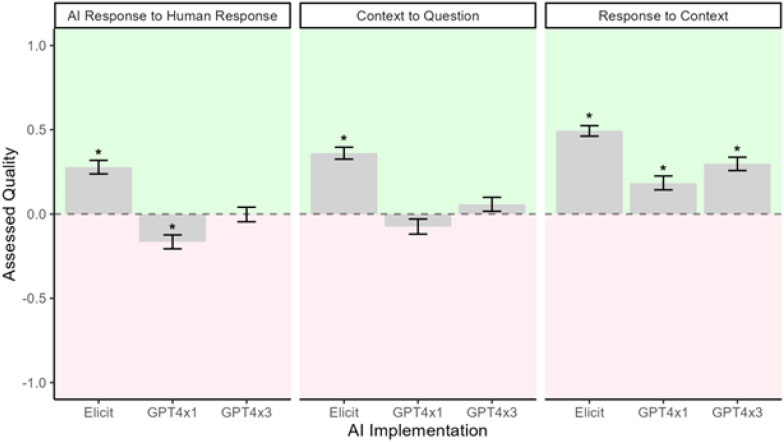
Mean assessed quality of different LLM evaluations. The data presented consolidates scores for (i) comparison of LLM and human responses, (ii) relevance of context to the question, and (iii) accuracy of the response in reflecting the context. On average, Elicit generally outperformed those of the GPT iterations, indicating a better alignment with the quality standards set by human evaluators. Asterisks represent statistically significant differences from a value of 0

In assessing the quality of LLM extractions, both human and LLM evaluators rated LLM responses on a three-point scale: −1 representing ‘poor’, 0 for ‘fair’, and 1 as ‘good’, across three criteria: (i) how the overall Response and Context compared to the human standard; (ii) how well the Context responded to the Question; (iii) how well the Response reflected the Context (Fig. 5). An extraction score above 0 was considered the threshold for acceptable quality. Notably, while LLMs sometimes failed to find relevant information (as shown in Fig. 4), when they did extract information, it was consistently accurate with essentially no instances of hallucination or factually incorrect content.

We employed a one sample t-test to examine the qualitative differences between the LLM extractions and the human-established acceptable quality threshold. Specifically, we used a two-sided alternative hypothesis to determine whether the LLM’s extraction quality was statistically significantly different from the score of 0, which represents our benchmark for ‘fair’ quality. The results for the first criterion (LLM Response compared to Human response) showed that GPT4x1’s mean extraction quality was significantly lower than the threshold (t = −4.00, p < 0.001), suggesting that its performance was not up to the acceptable standard. Conversely, Elicit exhibited a mean quality score significantly higher than the threshold (t = 6.91, p < 0.001), indicating a generally acceptable level of performance. GPT4x3’s quality was not statistically different from the threshold.

We also included an LLM evaluator (GPT4-Turbo, run 5 times per paper-question pair) in the assessment process to parallel the human evaluation [see Additional file 1]. Although the LLM evaluator tended to rate the responses more favorably than human evaluators, a linear mixed-effects model, assessed using ANOVA, showed that this difference was not statistically significant (p = 0.097), and broadly followed the same trends as the human evaluators. The second criterion (Context to Question) reflected similar trends to the first, with Elicit outperforming the GPT implementations. All three implementations satisfactorily provided meaningful Responses based on the provided Contexts.

We also analyzed the potential influence of the extractors themselves on the assessed quality using the same t-test and found no significant effect (p = 0.537). This suggests that the variation in extraction quality is attributable to the LLM systems rather than the individual human evaluators. Both human evaluators and LLM evaluators consistently rated Elicit as providing the highest quality responses, followed by GPT4x3 [see Additional file 1].

To further explore the differences between the LLM implementations, we utilised a linear mixed model, to account for both fixed effects (such as the specific LLM being used) and random effects (such as variability among papers or questions; [see Additional file 2]). We found significant differences in the quality of extractions produced by the LLM systems. Elicit performed the best, significantly outperforming both GPT4x1 (p < 0.001) and GPT4x3 (p < 0.001), indicating that its extractions were closer to the quality considered acceptable by human reviewers. Additionally, GPT4x3 was found to be significantly better than GPT4x1 (p = 0.006), suggesting that the iterative approach of running GPT4-Turbo multiple times yielded improved results over a single run.

### Manual review of flagged paper-question pairs

To ensure the validity of our findings and further understand the discrepancies between LLM and human extractions, we conducted a manual investigation of 56 paper-question pairs. These paper-question pairs were flagged by the independent evaluation team during their assessment, and were raised for a variety of reasons, including the need for additional context from the full-text to ensure a fair evaluation, or instances where there was a stark contrast between human and LLM responses.

Upon re-evaluation, we found that in 28 cases, the LLM provided appropriate responses that faithfully represented the content of the study, often with more detail or specificity than the human extractor. This included instances where the human extractor missed certain details, for instance, failing to report all the countries mentioned in a paper, which the LLM correctly identified. However, there were also 7 instances where the LLM clearly misinterpreted the question, leading to misrepresentations of the study content. One example being the fifth example in Table 2 where the human extractor found no relevant answer, whilst the LLMs provided convincing, yet misinterpreted responses. The remaining 21 outputs presented a challenge in classification; they contained truthful content that was related to the question but was too general or broad, potentially making them less useful for targeted queries. For example, in one such case, when asked to report the country of the case study, GPT4x1 reported ‘the South Pacific including Solomon Islands’. In another case, Elicit provided correct information about how the community monitors their resources, but also included several other passages of context that were not relevant to the question.

**Table 2:** Examples of extraction questions with human and LLM responses (longer responses are excerpted and indicated with [...])

Paper citation	Extraction question	Human response	GPT4x3	GPT4x1	Elicit
[37]	Which country was the study conducted in?	Solomon Islands	The study was conducted in Solomon Islands	The study was conducted in Roviana Lagoon, Solomon Islands.	Solomon Islands
[38]	What management mechanisms are used?	Creation of very small village-based marine reserves in which fishing is banned	Management mechanisms used include the creation of very small village-based marine reserves (tabu areas) with permanent or temporary fishing bans for certain invertebrate species and the translocation of middle-size and large trochus by local villagers to enhance population recovery within these protected zones. [...] Some common actions within these initiatives involve partial or total fishing ground closures, usually corresponding to very restricted parts of the reefs	No data	The management mechanisms used in the study are: - Creation of village-based marine reserves (tabu areas) with total fishing bans. - Translocation actions by local fishers to move certain species into protected zones to avoid poaching and increase future harvestable stock
[39]	How does the community monitor the system they are managing?	1. Indiscernible 2. Indiscernible 3. Survey 4. Indiscernible 5. Indiscernible	The community monitors the system they are managing through various marine resource management methods including enforcing restrictions on fishing methods, conducting fish stock surveys, and initiating marine protected areas. [...] Some communities also establish data gathering procedures to evaluate the effectiveness of their conservation efforts on marine resources such as the health of protected clam stocks	The community is using various practices to monitor the system they are managing, including enforcing measures and restrictions on fishing methods, conducting fish stock surveys and initiating marine protected areas. [...] An experiment in Fiji indicated that the local communities were able to monitor the impact of their management measures by conducting systematic data gathering regarding the health of protected clam stocks	The communities described in the paper monitor their marine resource management systems through direct observation and data collection by trained community members, as well as by integrating traditional observational methods with science-based techniques. [...]
[40]	What are the reported barriers to success of Community-Based Fisheries Management?	Poaching	The reported barriers to the success of Community-Based Fisheries Management are inaccurate local knowledge regarding spawning seasons, vulnerability of fish spawning aggregations to nighttime spearfishing, limited protection offered by the proposed closed season, and poaching despite the establishment of a no-take Marine Protected Area (MPA)	No data	Poaching by nighttime spearfishermen - Multiple communities claiming customary rights to fish the FSA, leading to difficulties in reaching management consensus - The remote location of the FSA, reducing the likelihood of detecting poaching activities - Limited capacity of local leaders and provincial fisheries officers to enforce the MPA closure
[37]	How does the community monitor the system they are managing?	No answer	The community, namely the fishers in Roviana Lagoon, Solomon Islands, monitors the system they are managing by carefully observing long-term ecological changes, specifically in the seagrass meadows. They detect shifts in the spatial coverage of these meadows and understand the driving factors behind these alterations. [...]	The fishers in Roviana Lagoon, Solomon Islands have shown the ability to effectively monitor long-term ecological changes in their environment, particularly in relation to seagrass meadows. They can detect changes in the spatial cover of the seagrass meadows and understand how these changes are driven. [...] Local resource users’ abilities to monitor these ecological changes shape marine resource use and practices, which can increase the management adaptability of indigenous or hybrid governance systems	The community in Roviana Lagoon monitors their marine resources through a combination of semi-structured interviews, group interviews with local ecological experts, historical aerial photography, IKONOS satellite images, and field-based surveys. [...]
[41]	How was the data on benefits collected?	Using genetic analysis to determine if protected MPA is benefiting community which created MPA	The data on benefits was collected through a genetic parentage analysis to ascertain larval dispersal from a single fish spawning aggregation of squaretail coral grouper at Manus, Papua New Guinea. [...] Adult coral grouper from a managed spawning aggregation were sampled, tagged, and tissue samples were obtained	The data on benefits, in this case the dispersal and contribution of larvae from a single fish spawning aggregation to fisheries replenishment, was collected using genetic parentage analysis. [...]	The method used to collect data on benefits was genetic parentage analysis, which involved sampling, measuring, and tagging adult coral groupers, as well as collecting and measuring juveniles. [...]

Table 2 provides representative examples of extraction questions with responses from both human extractors and all three LLM implementations. These examples illustrate the varying quality and detail of responses across different papers and questions, showing cases where LLMs provide more comprehensive information than humans, instances where different LLM implementations produce varying quality responses to the same question, and situations where some LLMs return “NO DATA” while others successfully extract relevant information.

### Response length analysis

Analysis of response lengths revealed significant differences in verbosity between human and LLM responses (one-way ANOVA, F = 130.86, p < 0.001). Human responses averaged 85 words (n=301, SD=82), while all LLM implementations produced significantly longer responses. Elicit generated the most verbose responses, averaging 306 words (n=396, SD=161), followed by GPT4x3 with 269 words (n=386, SD=213), and GPT4x1 with 187 words (n=283, SD=116).

Pairwise comparisons with Bonferroni correction ($\alpha$ = 0.0083) showed that all differences between implementations were statistically significant. All LLM implementations produced significantly longer responses than humans (p < 0.001 for all comparisons). Among the LLM implementations, Elicit’s responses were significantly longer than both GPT4x3 (p = 0.006) and GPT4x1 (p < 0.001), and GPT4x3’s responses were significantly longer than GPT4x1 (p < 0.001).

To examine whether response length influenced extraction quality, we analyzed the correlation between response length and quality scores across all LLM implementations (n=966). We found a very weak positive correlation (r = 0.067, p = 0.037) between response length and quality. Analysis by length ranges revealed that responses between 101–200 words achieved the highest mean quality score (2.30), while very short responses (0–100 words) received the lowest mean quality score (1.89). However, increasing length beyond 200 words did not lead to further improvements in quality, with responses over 400 words averaging similar quality scores (2.09) to shorter responses. This suggests that while extremely concise responses may be suboptimal, there is no clear benefit to the high verbosity observed in many LLM responses.

### Drivers of LLM extraction quality

We found that the assessed quality scores were not associated with question content (p = 0.067). We noted differences in the quality of responses across different questions, although amongst the factors that we investigated, we did not find any strong evidence to draw conclusions about what drives these differences. Despite there being no overall effect of the question on the assessed quality values, we did find three pairs of questions were significantly different in terms of the evaluated qualities. These were Q1 (‘Which country was the study conducted in?’) vs. Q2 (‘Provide some background as to the drivers and/or motivators of community-based fisheries management.’) (p < 0.001), Q1 vs. Q7 (‘How was the data on benefits collected?’) (p < 0.001), and Q3 (‘What management mechanisms are used’) vs. Q9 (‘Guidelines for future implementation of CBFM?’) (p < 0.001). The rest of the pair-wise evaluations were not statistically different.

**Fig. 6:**
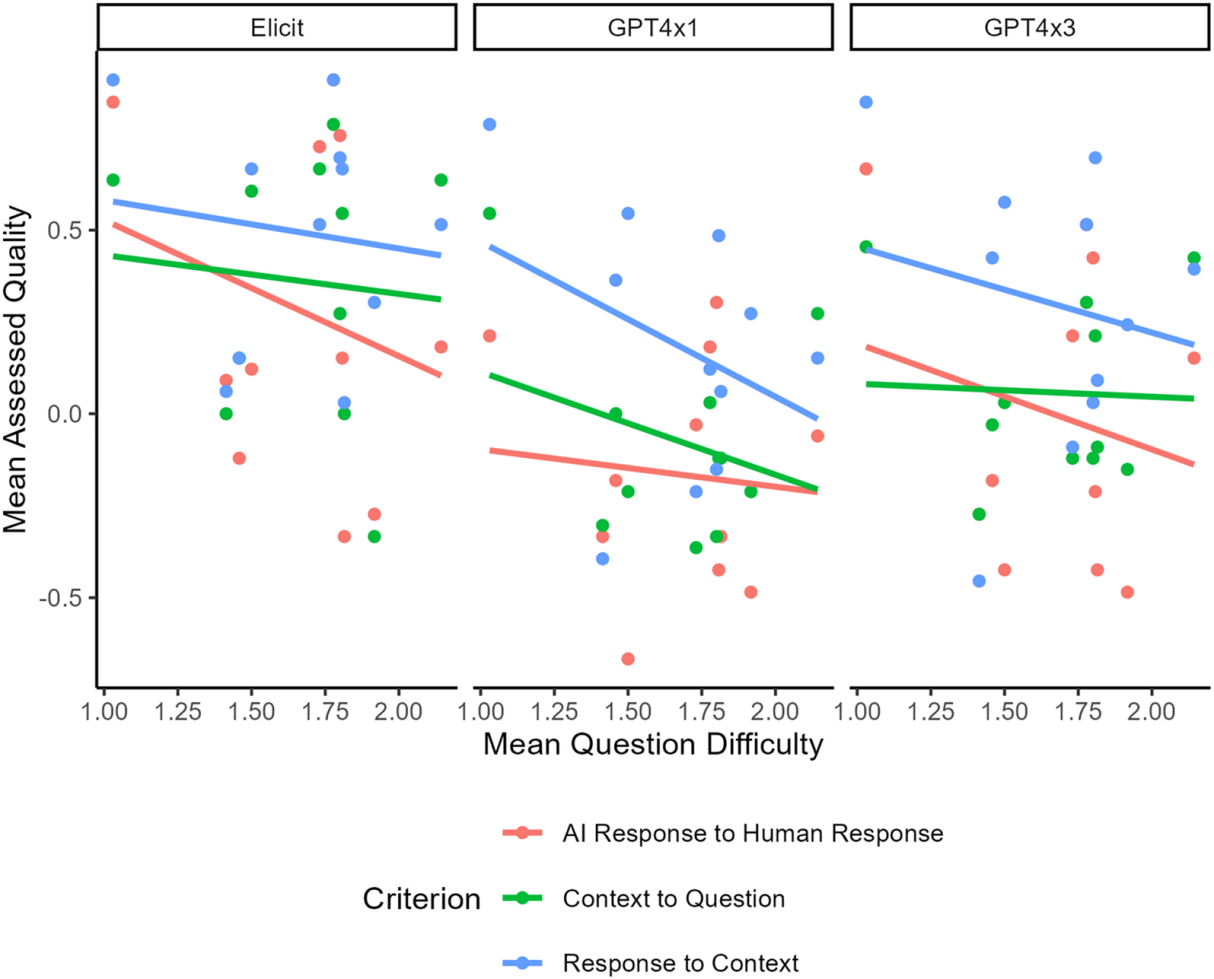
Comparison of LLM performance across three implementations (Elicit, GPT4x1, and GPT4x3) in answering questions about scientific literature. The graph plots Mean Assessed Quality against Mean Question Difficulty for three evaluation criteria: LLM Response to Human Response, Context to Question, and Response to Context. Each point represents the mean question difficulty across all papers for a specific criterion and question. Trend lines illustrate the relationship between question difficulty and assessed quality for each criterion. The x-axis (Mean Question Difficulty) shows the average difficulty rating assigned by humans to each question, ranging from 1.0 to 2.0. The y-axis (Mean Assessed Quality) indicates how well the LLM performed on the tasks, as judged by human evaluators

We found that the assessed quality of the LLM responses was not significantly related to difficulty (p = 0.055) of the paper-question pair, as ranked by the human extraction team, nor was there a significant interaction between the difficulty and the LLM implementation (p = 0.877) (Fig. 6). Therefore, regardless of the difficulty of the paper-question pair, the mean assessed quality was not affected for any of the LLM implementations.

It is important to note that these results are based on a limited sample of 33 papers in the specific domain of community-based fisheries management. While they provide valuable insights into the performance of LLMs in this context, further research with larger and more diverse datasets is needed to confirm the generalizability of these findings to other fields and types of literature.

## Discussion

### How well can LLMs discern the presence or absence of relevant contextual data?

Our results provide evidence that specialised text-extraction tools like Elicit, and general large language models like GPT4-Turbo, can facilitate, but not reliably automate, the extraction stage of evidence synthesis in the context of community-based fisheries management (CBFM) literature. Among the LLM implementations we tested, Elicit consistently provided higher quality responses compared to our implementations based on GPT4-Turbo, and also had a negligible false negative rate, which means it was much less prone to missing relevant information. We also showed that relying on a generalist LLM like GPT4-Turbo may not be immediately effective, but its performance can be improved through aggregating repeated identical calls.

However, the LLMs we tested were not reliable enough to be trusted to perform the task without human involvement. While Elicit outperformed the other tools tested, its responses tended to include a large quantity of unnecessary extra information in addition to the relevant material. In the context of evidence synthesis, this is preferable to failing to deliver relevant information but means that additional human work is still required to ’sift out’ the relevant material from responses. Our findings contrast with recent studies on LLM-based scientific information extraction. For example, Gougherty & Clipp [24] found that LLMs can achieve very high accuracy (>90%) when extracting discrete and categorical data from ecological literature, though they noted similar challenges with certain types of quantitative information. On an even larger scale, Keck et al. [25] demonstrated similar performance (70% sensitivity, 89.5% precision) when extracting ecological interaction data from nearly 84,000 scientific articles. Meanwhile, Dagdelen et al. [22] showed that fine-tuned LLMs can successfully extract complex scientific relationships when given well-defined schemas. Our lower rates of agreement likely reflect the more challenging nature of extracting qualitative contextual information that requires synthesis and interpretation across full papers, compared to the more structured extraction tasks in these studies.

While our study focused on papers containing relevant data for extraction, it’s worth noting that we found near-zero rates of hallucination in the LLM responses (only one instance in 29 flagged contexts). This suggests that false information generation may not be a major concern for these tools in this context, though future work could specifically examine their behavior with papers lacking relevant information. Our focus on papers with extractable data was driven by our primary research goal of evaluating extraction quality in the context where these tools would be most commonly applied—synthesizing information that exists in the literature.

### How does the quality of LLM extraction outputs compare to those done by human researchers?

In the context of actually deploying these tools in a systematic evidence synthesis for CBFM, our findings suggest that human oversight is still important in this extraction stage of the process. However, we also show that in some cases the LLM responses were in fact better than the human responses, either due to level of detail or the ability to find relevant information.

The implications of our findings indicate a future collaborative approach to evidence synthesis with LLMs in the field of CBFM. By demonstrating that LLMs can sometimes outperform humans in terms of detail and information retrieval, our research suggests that these tools have the potential to enhance the breadth and depth of systematic reviews in this domain. While LLM-based approaches could support reproducibility through standardised extraction procedures, it’s important to note that the non-deterministic nature of current LLMs and their temperature settings means that exact reproduction of results may not be possible. However, the general patterns and capabilities we’ve identified should remain consistent across runs.

While we acknowledge the limitations of studying proprietary LLMs whose underlying models may change over time, we believe this research provides valuable insights for the scientific community. These tools are already being widely adopted by researchers for evidence synthesis tasks, making it crucial to understand their current capabilities and limitations. By documenting their performance at this point in time, we provide important benchmarks against which future improvements can be measured. Additionally, our findings about the strengths and weaknesses of different approaches (e.g., Elicit’s lower false negative rate but tendency toward verbose responses) can help inform best practices for their use, even as the specific tools continue to evolve.

### Does the difficulty or type of question influence the performance of the extraction?

Our finding that question difficulty, as rated subjectively by the human extractors, was not strongly correlated with the quality of LLM outputs provides evidence to suggest that these tools can perform well with CBFM documents that might be otherwise difficult for humans to extract information from. However, we note that in the case of a human-driven review, there is a learning process that emerges from attempting to apply one’s analytical schema to a body of literature, failing, and then adapting, which is a key element of formal methodologies in this space [28, 42, 43]. This can lead to an iterative refinement of the research questions and goals, which would not be possible under the LLM-enabled paradigm that we have tested here.

This suggests that when undertaking these kinds of systematic reviews, collaborative approaches between humans and LLMs may prove to be more fruitful than compared to each working in isolation [44, 45]. While LLMs aren’t perfect at data extraction tasks, the speed and level of accuracy they achieve can add strength to a review, similar to how having redundant human reviewers ensures important details aren’t missed. The trade-off involves the number of false positives researchers would need to weed out, though it’s worth noting that disagreement and mistake-making also occur between human researchers, and multiple reviewers examining the same articles is generally recognized as valuable.

We imagine that there could be a number of ways to improve and develop such a collaborative workflow to improve the quality of evidence syntheses. In line with practice in this study, LLMs could be deployed independent of a human extraction, and both datasets could be combined to produce a synthesis. Adding one or a diversity of LLM reviewers in this way could potentially serve to reduce the bias present in a review by providing additional ’perspectives’ to those that the human reviewer team has, or as an additional coder to calculate intercoder reliability when undertaking a review alone. Or alternatively, LLMs could be implemented as the first stage of the synthesis and then the human could proof the outputs and/or cross-check a sample of data points. In this approach, human reviewers could use the near-zero rate of false negatives (i.e., returning responses to all questions) as a starting point to manually eliminate the false positives.

The decision to adopt LLM-assisted versus human-only approaches should consider the scale of the review. For large reviews with hundreds of papers, the time savings from using LLMs for initial extraction may outweigh verification costs, making them particularly valuable as a second source of validation to human-driven extraction. For smaller, specialized reviews, traditional human extraction might still be more efficient given the overhead of verifying LLM outputs.

Future work could explore a range of workflows in a rigorous way to identify promising best practices that prioritise the quality and precision of the overall extraction procedure. We note that as LLMs continue to improve in capabilities, it will be important to continuously benchmark tools—perhaps automatically [46]. And whilst general benchmarks for text extraction exist, the nuances of contextual understanding present in the field of environmental management, and the importance of fully reckoning with the complexities of environmental systems, will likely require domain specific benchmarks.

### Limitations and future work

While our pilot study demonstrates the potential of LLMs in extracting qualitative data from CBFM literature, it is crucial to acknowledge several important limitations. The narrow focus on community-based fisheries management literature, while allowing for in-depth insights, constrains the generalizability of our findings to other fields of study. This limitation is further compounded by our relatively small sample size of 33 papers, which impacts the statistical power of our analysis and the broader applicability of our results. However, this challenge of limited sample sizes is likely to be encountered in many environmental and conservation topics, particularly when studying emerging or niche areas. The relative scarcity of literature in specialised environmental topics means that LLM performance on these subjects may be inherently limited, both by the small number of papers available for testing and by the potentially limited representation of these topics in LLM training data. This suggests that particular care should be taken when applying LLMs to novel or specialised environmental topics, as the models may struggle with concepts and terminology that are underrepresented in their training data.

The performance of LLMs observed in our study may be influenced by characteristics specific to CBFM literature. There could be discipline-specific biases in language use, structure, or content that affect LLM performance, which might not be present or might manifest differently in other fields. Consequently, caution must be exercised when extrapolating our results to other disciplines or larger datasets, as the effectiveness of LLMs in qualitative data extraction may vary significantly across different subject areas or with more diverse types of literature.

The proprietary nature of the tools used in our study (Elicit and GPT4-Turbo) raises concerns about reproducibility and widespread adoption. The performance of these tools may not be consistent across different versions or implementations, potentially limiting the replicability of our findings. Furthermore, unlike human-driven reviews, our LLM-enabled approach did not allow for the iterative refinement of research questions and goals that often occurs during the review process. This limitation could impact the depth and nuance of the extracted information, as the adaptive nature of human-led reviews was not fully replicated in our LLM-assisted methodology. Additionally, our study tested only one semantic construction per question, which may not fully capture the capabilities of these models. LLMs have shown sensitivity to slight changes in question phrasing, meaning that different formulations of the same question could yield varying results. Future work should explore the impact of varied question formulations on extraction quality and develop best practices for question construction.

Our evaluation of LLM performance relied partly on subjective assessments by human experts, which could introduce bias into our results. Notably, a methodological limitation of our study was the non-blind evaluation process, where evaluators knew whether extractions came from humans or LLMs. Future studies should implement fully blind validation protocols where evaluators cannot distinguish between human and LLM-generated extractions, which would provide more objective assessments of extraction quality. Additionally, the rapid evolution of these models, especially those like GPT4-Turbo, means that our results reflect the capabilities of these tools at the time of the study and may not represent their current or future performance. This temporal limitation underscores the need for ongoing assessment of LLM capabilities in the context of evidence synthesis. There is also a risk that widespread adoption of these tools could lead to homogenization of literature interpretations, as different research teams might receive similar LLM-generated extractions rather than developing their own unique perspectives through deep engagement with the literature. This reinforces the importance of maintaining human oversight and encouraging diverse analytical approaches.

In topics with strong components of equity, local and traditional knowledge, or ethical considerations—which are often present in CBFM literature—there is a higher risk of LLMs misinterpreting or oversimplifying complex cultural or contextual information. This potential for misinterpretation highlights the continued importance of human oversight in LLM-assisted research, particularly in fields dealing with sensitive or nuanced information.

Based on these limitations, several directions for future research emerge. Expanding the study to encompass multiple disciplines and larger sample sizes would be important to verify the generalizability of our findings. Such scaling efforts would benefit from testing additional state-of-the-art LLMs to provide a more comprehensive evaluation of available tools. The selection of models for larger-scale studies should prioritise those with documented stability in their APIs, clear versioning, and established track records in academic applications. Additionally, testing both open-source and proprietary models would help address reproducibility concerns while providing insights into the relative capabilities of different approaches. Investigating how LLM performance varies across different fields of study beyond CBFM, particularly in areas with different structural or linguistic characteristics, would provide valuable insights into the broader applicability of these tools in evidence synthesis.

Future work should also explore the impact of document complexity, writing style, and discipline-specific jargon on LLM extraction quality. This could lead to the development of more nuanced tools capable of handling diverse types of academic literature. Additionally, research into methods for incorporating iterative refinement processes into LLM-assisted literature reviews could help mimic the adaptive nature of human-driven reviews, potentially enhancing the depth and accuracy of extracted information.

Longitudinal studies assessing how the performance of LLMs in qualitative data extraction changes over time would be valuable as these technologies continue to evolve. Exploring open-source alternatives could address concerns about the accessibility and reproducibility of LLM-assisted research methods, potentially broadening the adoption of these techniques in the academic community.

Finally, examining the ethical implications of using LLMs in evidence synthesis, particularly in fields where misinterpretation could have significant consequences, is a critical area for future research. This could lead to the development of guidelines and best practices for the responsible integration of these tools into research methodologies across various disciplines.

In conclusion, while our study demonstrates the potential of LLMs in extracting qualitative data from CBFM literature, it also underscores the need for continued human oversight and further research. The integration of LLMs into evidence synthesis processes holds promise for enhancing efficiency and comprehensiveness, but it must be approached with an awareness of their current limitations and potential biases. Future work should focus on addressing these limitations and developing robust methodologies for LLM-assisted evidence synthesis across diverse fields of study, always maintaining a balance between technological innovation and the nuanced understanding that human researchers bring to the process.

## Supplementary Information

Supplementary material 1. Extended Results ($AF1_Extended.pdf$). Contains a comprehensive list of papers included in the review and extended results including figures showing GPT-4 quality assessment and mean quality of AI responses for each question.

Supplementary material 2. Statistical Analysis ($AF2_Statistical.pdf$). Contains detailed statistical analysis including t-tests, linear mixed-effects models, and ANOVA results examining the effects of different AI implementations, question types, difficulty levels, and assessor types on extraction quality.

## Data Availability

All of the supporting code and data are available at the GitHub repository: https://github.com/s-spillias/AI_Extraction.
