# Supplementary material for: Evaluating generative AI for qualitative data extraction in community-based fisheries management literature
Source: Environ Evid. 2025 Jun 2;14:9. doi: 10.1186/s13750-025-00362-9 (PMC12128520; doi:10.1186/s13750-025-00362-9)
Supplement: Supplementary file 1 — Supplementary material 1. Extended Results ($AF1_Extended.pdf$AF1Extended.pdf). Contains a comprehensive list of papers included in the review and extended results including figures showing GPT-4 quality assessment and mean quality of AI responses for each question. [file 13750_2025_362_MOESM1_ESM.pdf]

Statistical Analysis for ‘Evaluating Generative AI to Extract Qualitative Data from Peer-Reviewed Documents’

2024-07-13

```r
# Initializing file paths and reading various datasets for analysis.
# The datasets include AI and human annotations, as well as validation assessments.

# File path for the main dataset.
file_path = 'Data/output_assessment_CHATGPT_explanation.xlsx'
# List of validation file paths.
validate_path = list.files('Data/Raw_Team_Assessment', pattern = 'validate', full.names = TRUE)
# Path to store figures.
figure_folder = '../Manuscript/Figures'

# Reading all sheets from the main Excel file and creating a list of dataframes.
sheet_names <- excel_sheets(file_path)
all_data_list <- lapply(sheet_names, function(sheet) {
  read_excel(file_path, sheet = sheet) %>%
    mutate(AI = sheet)})

# Defining a function to calculate summary statistics.
do_stats = function(x){
  x %>%
    summarise(mean = mean(value),
              standard_error = sd(value, na.rm = TRUE) / sqrt(n()),
              st_dev = sd(value),
              difficulty_mean = mean(Difficulty, na.rm = TRUE),
              st_dev_difficulty = sd(Difficulty, na.rm = TRUE),
              standard_error_difficulty = sd(Difficulty, na.rm = TRUE) / sqrt(n()),
    )}

# Reading and processing difficulty levels from the human extraction dataset.
difficulty <- read_excel("Data/Extraction_Human_Difficulty.xlsx") %>% as_tibble() %>%
  pivot_longer(-Question) %>%
  setNames(c('Citation','Question','Difficulty')) %>%
  mutate(Difficulty = case_match(Difficulty, 'Easy' ~ 1, 'Medium' ~ 2, 'Hard' ~ 3))

file_path = 'Data/Extraction_ai.xlsx'
sheets = excel_sheets(file_path)
ai_extractions <- sheets %>% lapply(function(x) read_excel(file_path, sheet = x) %>% as_tibble() %>%
                                      mutate(across(everything(), function(x) x %>% str_replace("Respons
                                      separate_wider_delim(-Citation, delim = "Context:", names = c('Re
                                      mutate(across(-Citation, nchar))) %>% setNames(sheets)
# Read and process the human extraction data from the Excel file.
human_extraction = "Data/Human_Extraction_Cleaned.xlsx" %>%
  read_excel(sheet = 'Human') %>%
  select(-5, -(seq(8, ncol(.), by = 3))) %>%
```

```
  select(-1) %>%
  slice(-(1:2)) %>%
  mutate(across(-Question, nchar)) %>%
  select(-last_col())
```

```
## New names:
## * `` -> `...4`
## * `` -> `...5`
## * `` -> `...7`
## * `` -> `...8`
## * `` -> `...10`
## * `` -> `...11`
## * `` -> `...13`
## * `` -> `...14`
## * `` -> `...16`
## * `` -> `...17`
## * `` -> `...19`
## * `` -> `...20`
## * `` -> `...22`
## * `` -> `...23`
## * `` -> `...25`
## * `` -> `...26`
## * `` -> `...28`
## * `` -> `...29`
## * `` -> `...31`
## * `` -> `...32`
## * `` -> `...34`
## * `` -> `...35`
## * `` -> `...36`
```

```
# Calculate verbosity ratios by comparing the length of AI and human extractions.
verbosity_ratios = sheets %>% lapply(function(x) bind_cols(human_extraction$Question, (ai_extractions[[
```

```
## New names:
## New names:
## New names:
## * `` -> `...1`
```

```
# Define the list of reviewers and corresponding questions for assessment.
reviewers = c(
  'R1', "Which country was the study conducted in?",
  'R1',"Provide some background as to the drivers and/or motivators of community-based fisheries managem
  'R2',"What management mechanisms are used?",
  'R3',"Which groups of people are involved in the management as part of the CBFM case-studies? Choices
  'R4',"What benefits of Community-Based Fisheries Management are reported in this case study?",
  'R4',"What are the indicators of success of CBFM?",
  'R1',"How was the data on benefits collected?",
  'R3',"What are the reported barriers to success of Community-Based Fisheries Management?",
  'R2',"Guidelines for future implementation of CBFM?",
  'R3',"How does the community monitor the system they are managing?",
  'R4',"How does the community make decisions?")

# Read and process the human extraction data, creating an 'extractor' data frame with relevant columns.
# The citation column is cleaned to create a shortened citation for easier handling.
extractor <- "Data/Human_Extraction_Cleaned.xlsx" %>%
```

```
  read_excel(sheet = 'Human') %>%
  dplyr::select(-5, -(seq(8, ncol(.), by = 3))) %>%
  #dplyr::select(-1) %>%
  dplyr::slice(-(1:2)) %>%
  mutate(across(-Question, nchar)) %>%
  dplyr::select(-last_col()) %>%
  select(-3:-24) %>%
  mutate(short_cit = substr(Question, 1,35)) #no idea why citation column is actually the question colu
```

```
## New names:
## * `` -> `...4`
## * `` -> `...5`
## * `` -> `...7`
## * `` -> `...8`
## * `` -> `...10`
## * `` -> `...11`
## * `` -> `...13`
## * `` -> `...14`
## * `` -> `...16`
## * `` -> `...17`
## * `` -> `...19`
## * `` -> `...20`
## * `` -> `...22`
## * `` -> `...23`
## * `` -> `...25`
## * `` -> `...26`
## * `` -> `...28`
## * `` -> `...29`
## * `` -> `...31`
## * `` -> `...32`
## * `` -> `...34`
## * `` -> `...35`
## * `` -> `...36`
```

```
colnames(extractor)[1] <- "Extractor"
colnames(extractor)[2] <- "Citation"
colnames(extractor)[3] <- "short_cit"

# Create a tibble of reviewers and their associated questions for later use in the analysis.
reviewers_id <- matrix(reviewers, nrow = length(reviewers) / 2, byrow = TRUE) %>% as_tibble() %>%
  setNames(c("Reviewer","Question"))
```

```
## Warning: The `x` argument of `as_tibble.matrix()` must have unique column names if
## `.name_repair` is omitted as of tibble 2.0.0.
## i Using compatibility `.name_repair`.
## This warning is displayed once every 8 hours.
## Call `lifecycle::last_lifecycle_warnings()` to see where this warning was
## generated.
```

```
# Combine all data sheets into one data frame, 'df_description', for further analysis.
df_description = all_data_list %>% bind_rows() %>%
  pivot_longer(-c('Citation','AI')) %>%
  separate(value, sep = ':::', into = c("value", "description")) %>%
  separate(value, sep = "\\|", into = paste0("rep",1:5)) %>%
  separate(description, sep = "\\|", into = paste0("Description",1:5)) %>%
```

```r
  pivot_longer(starts_with('rep'), names_to = 'Agent')
```

```
## Warning: Expected 2 pieces. Missing pieces filled with `NA` in 1 rows [760].
```

```r
# Further process 'df_description' to create 'ai_df' by separating criteria and pivoting longer for ana
ai_df <- df_description %>%
  dplyr::select(-starts_with('Description')) %>%
  separate(value, sep = ';', into = paste0('Crit', 1:4)) %>%
  pivot_longer(starts_with('Crit'), names_to = 'Criteria')
```

```
## Warning: Expected 4 pieces. Missing pieces filled with `NA` in 5445 rows [1, 2, 3, 4, 5,
## 6, 7, 8, 9, 10, 11, 12, 13, 14, 15, 16, 17, 18, 19, 20, ...].
```

```r
# Read team assessment data from the specified path and create a list of data frames, 'team_data_list'.
team_path = "Data/output_assessment_TEAM.xlsx" #original file with unedited flag values = output_assess

sheet_names <- excel_sheets(team_path)

team_data_list <- lapply(sheet_names, function(sheet) {
  read_excel(team_path, sheet = sheet) %>%
    mutate(AI = sheet)
})

# Process the validation files to create a unified 'df_validate' data frame.
# This involves reading the data, binding rows, and reshaping the data frame for analysis.


stat_summary <- team_data_list %>% bind_rows() %>%
  pivot_longer(-c("Citation","AI")) %>%
  separate(value, sep = ';', into = paste0('Crit',1:4)) %>%
  pivot_longer(starts_with("Crit"), names_to = 'Criteria') %>%
  mutate(Agent = 'Human') %>%
  bind_rows(ai_df) %>%
  mutate(Flag = ifelse(Criteria == 'Crit4' & value > 1, "FLAG",NA),
         group_agent = ifelse(str_detect(Agent, "Human"), "Human Assessor", "GPT4-Turbo Assessor"),
         value = as.integer(value)) %>%
  {flag_df <<- .} %>%
  filter(!is.na(value)) %>%
  filter(Criteria != 'Crit4') %>%
  relocate(value, .after = 'group_agent') %>%
  mutate(Criteria = case_match(Criteria, 'Crit1' ~ 'Context to Question',
                               'Crit2' ~ 'Response to Context',
                               'Crit3' ~ 'AI Response to Human Response')) %>%
  rename('Question' = 'name') %>%
  left_join(reviewers_id, by = 'Question') %>%
  mutate(Reviewer = ifelse(str_detect(group_agent,'GPT'),'GPT4-Turbo',Reviewer)) %>%
  mutate(short_cit = substr(Citation, 1,35)) %>%
  left_join(difficulty %>%  mutate(short_cit = substr(Citation, 1,35)) %>% dplyr::select(-Citation), by
  mutate(AI = case_match(AI, 'ELICIT' ~ 'Elicit',
                         'OPENAI' ~ 'GPT4x3',
                         'OPENAI_single' ~ 'GPT4x1')) %>%

  mutate(Question = factor(str_split_fixed(Question, '\\?',2)[,1],levels = str_split_fixed(reviewers_id
  {full_df <<- .} %>%
  group_by(group_agent, AI,Criteria) %>%
```

```r
  do_stats()
```

```
## Warning: Expected 4 pieces. Missing pieces filled with `NA` in 659 rows [1, 3, 4, 5, 6,
## 8, 9, 11, 12, 14, 15, 16, 17, 19, 20, 22, 23, 25, 26, 27, ...].

## Warning: There was 1 warning in `mutate()`.
## i In argument: `value = as.integer(value)`.
## Caused by warning:
## ! NAs introduced by coercion

## `summarise()` has grouped output by 'group_agent', 'AI'. You can override using
## the `.groups` argument.
```

```r
full_df <- full_df %>%
  left_join(extractor, by = "short_cit") %>%
  mutate(Extractor = ifelse(group_agent == "Human Assessor", Extractor, "AI")) %>%
  select(-Citation.y) %>%
  rename(Citation = Citation.x)

question_df <- full_df %>%
  group_by(Reviewer, AI, Criteria, Question) %>%
  do_stats()
```

```
## `summarise()` has grouped output by 'Reviewer', 'AI', 'Criteria'. You can
## override using the `.groups` argument.
```

```r
paper_df <- full_df %>%
  group_by(group_agent, AI, Criteria, Citation) %>%
  do_stats()
```

```
## `summarise()` has grouped output by 'group_agent', 'AI', 'Criteria'. You can
## override using the `.groups` argument.
```

```r
reviewer_df <- full_df %>%
  group_by(Reviewer, Criteria) %>%
  do_stats()
```

```
## `summarise()` has grouped output by 'Reviewer'. You can override using the
## `.groups` argument.
```

```r
flag_df <- flag_df %>% filter(!is.na(Flag))

full_df$value <- full_df$value - 2

humandf <- full_df %>% filter(Agent == "Human") %>% select(-short_cit)
aidf <- full_df %>% filter(Agent != "Human")
aidf2 <- aidf %>% group_by(Citation, AI, Question, Criteria, Flag, group_agent, Reviewer, Difficulty, E
  summarise(value = median(value)) %>% mutate(Agent = "AI") %>%
  select(Citation, AI, Question, Criteria, Agent, Flag, group_agent, value, Reviewer, Difficulty, Extrac
```

```
## `summarise()` has grouped output by 'Citation', 'AI', 'Question', 'Criteria',
## 'Flag', 'group_agent', 'Reviewer', 'Difficulty'. You can override using the
## `.groups` argument.
```

```r
statdf <- rbind(humandf, aidf2)
statdfx <- statdf %>% filter(Agent == "Human")

diffdata <- paper_df %>% filter(group_agent != "GPT4-Turbo Assessor") %>% mutate(short_cit = substr(Cita
```

```r
diffdata <- diffdata %>% left_join(select(extractor, short_cit, Extractor), by = c("short_cit"))
diffdata <- subset(diffdata, select = -short_cit)
```

T-test: Comparing values to the mean of 0

Conducting a one-sample t-test to determine if the mean of the assessed values for AI Response to Human Response significantly differs from the hypothetical mean value of 0. This test helps us understand whether the AI's performance is above or below the baseline of no difference (0), which would indicate no response or a neutral response. We perform this test for each AI implementation to evaluate their individual performances.

```
## # A tibble: 3 x 3
##   AI      t_value  p_value
##   <chr>     <dbl>    <dbl>
## 1 Elicit   6.91   2.16e-11
## 2 GPT4x1  -4.00   7.60e- 5
## 3 GPT4x3  -0.0638 9.49e- 1
```

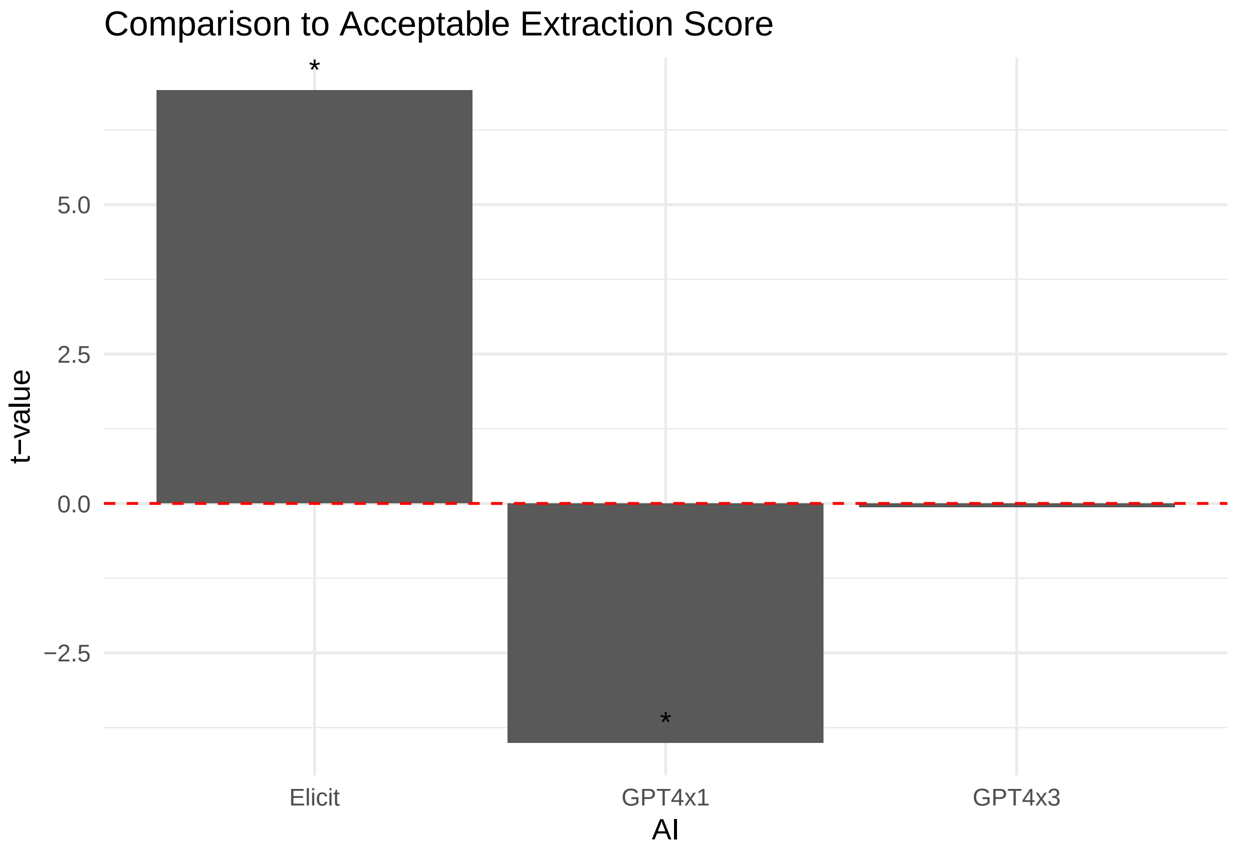

Effect of extractor on value and paper on value

Assessing the influence of the extractor (human or AI) on the mean assessed values. We use a linear model to compare the mean values across different annotators and determine if the source of extraction significantly affects the assessed values.

```
model_extractor <- lm(mean ~ Extractor, data = filter(diffdata, Criteria == "AI Response to Human Respo
anova(model_extractor)
```

```
## Analysis of Variance Table
##
## Response: mean
##           Df  Sum Sq  Mean Sq F value Pr(>F)
## Extractor  1  0.0497 0.049725  0.3825 0.5377
## Residuals 97 12.6101 0.130001
```

Effect of AI on values (AI response to human response)

Utilizing a linear mixed-effects model to examine the impact of different AI implementations on the assessed values of AI Response to Human Response. The model includes the AI as a fixed effect and both reviewer and citation as random effects to account for variability across different reviewers and papers.

```
aieffect <- lmer(value ~ AI + (1|Reviewer) + (1|Citation), data = filter(statdfx, Criteria == "AI Respo
summary(aieffect)
```

```
## Linear mixed model fit by REML. t-tests use Satterthwaite's method [
## lmerModLmerTest]
## Formula: value ~ AI + (1 | Reviewer) + (1 | Citation)
##    Data: filter(statdfx, Criteria == "AI Response to Human Response")
##
## REML criterion at convergence: 2428.4
##
## Scaled residuals:
##      Min       1Q   Median       3Q      Max
## -2.43377 -0.79938  0.08517  0.78605  2.18656
##
## Random effects:
##  Groups   Name        Variance Std.Dev.
##  Citation (Intercept) 0.04657  0.2158
##  Reviewer (Intercept) 0.08873  0.2979
##  Residual             0.51192  0.7155
## Number of obs: 1089, groups:  Citation, 33; Reviewer, 4
##
## Fixed effects:
##               Estimate Std. Error         df t value Pr(>|t|)
## (Intercept)    0.26583    0.15817    3.66456   1.681    0.175
## AIGPT4x1      -0.44353    0.05311 1051.01010  -8.351  < 2e-16 ***
## AIGPT4x3      -0.28099    0.05311 1051.01010  -5.291 1.48e-07 ***
## ---
## Signif. codes:  0 '***' 0.001 '**' 0.01 '*' 0.05 '.' 0.1 ' ' 1
##
## Correlation of Fixed Effects:
##          (Intr) AIGPT41
```

```
## AIGPT4x1 -0.168
## AIGPT4x3 -0.168  0.500
```

```r
anova(aieffect)
```

```
## Type III Analysis of Variance Table with Satterthwaite's method
##    Sum Sq Mean Sq NumDF DenDF F value    Pr(>F)
## AI 36.553  18.276     2  1051  35.702 9.973e-16 ***
## ---
## Signif. codes:  0 '***' 0.001 '**' 0.01 '*' 0.05 '.' 0.1 ' ' 1
```

Pairwise comparisons of the AI implementations are performed to understand which differences are significant.

Tukey's method is used for multiple comparisons to adjust for the possibility of type I error.

```r
aieffect_emmeans <- emmeans(aieffect, ~ AI)
aieffect_cont <- pairs(aieffect_emmeans, adjust = "tukey")
print(aieffect_cont)
```

```
##  contrast        estimate     SE   df t.ratio p.value
##  Elicit - GPT4x1    0.444 0.0531 1051   8.351  <.0001
##  Elicit - GPT4x3    0.281 0.0531 1051   5.291  <.0001
##  GPT4x1 - GPT4x3   -0.163 0.0531 1051  -3.060  0.0064
##
## Degrees-of-freedom method: kenward-roger
## P value adjustment: tukey method for comparing a family of 3 estimates
```

Effect of the individual questions on assessment values

Investigating the impact of individual questions on the assessed values using a linear mixed-effects model. This model allows us to see whether the content of the question affects the quality of AI responses, with question as a fixed effect and reviewer, citation, and AI as random effects to capture variation not explained by the questions alone.

```r
qeffect <- lmer(value ~ Question + (1|Reviewer) + (1|Citation) + (1|AI), data = filter(statdfx, Criteri
```

```
## Warning in checkConv(attr(opt, "derivs"), opt$par, ctrl = control$checkConv, : Model is nearly unide
##  - Rescale variables?
```

```r
anova(qeffect)
```

```
## Type III Analysis of Variance Table with Satterthwaite's method
##          Sum Sq Mean Sq NumDF DenDF F value  Pr(>F)
## Question 64.576  6.4576    10     2  14.222 0.06744 .
## ---
## Signif. codes:  0 '***' 0.001 '**' 0.01 '*' 0.05 '.' 0.1 ' ' 1
```

Effect of difficulty on values

Assessing how the difficulty level, as perceived by human annotators, influences the assessed quality of AI responses. A linear mixed-effects model is constructed with an interaction term between Difficulty and AI to explore if and how the difficulty of questions may affect AI performance differently. Citation, extractor, and reviewer are included as random effects to control for their potential confounding impacts.

```
diff <- lmer(value ~ Difficulty*AI + (1|Citation) + (1|Extractor) + (1|Reviewer), data = filter(statdfx
```

```
## boundary (singular) fit: see help('isSingular')
```

```
anova(diff)
```

```
## Type III Analysis of Variance Table with Satterthwaite's method
##                Sum Sq Mean Sq NumDF  DenDF F value   Pr(>F)
## Difficulty    1.8060 1.80600     1 806.24  3.6882 0.055152 .
## AI            5.1395 2.56977     2 776.34  5.2479 0.005447 **
## Difficulty:AI 0.1278 0.06391     2 776.34  0.1305 0.877665
## ---
## Signif. codes:  0 '***' 0.001 '**' 0.01 '*' 0.05 '.' 0.1 ' ' 1
```

Effect of assessor on assesed quality (GPT4 vs human)

Exploring the differences in assessed quality between GPT4-Turbo and human assessors. A linear mixed-effects model is used to analyze the effect of the group agent (GPT4 or Human) on the assessed values. This model accounts for the potential variability introduced by the individual annotators and reviewers, which are included as random effects, to isolate the effect of the assessor type.

```
assessor <- lmer(value ~ group_agent + (1|Extractor) + (1|Reviewer), data = full_df)
anova(assessor)
```

```
## Type III Analysis of Variance Table with Satterthwaite's method
##             Sum Sq Mean Sq NumDF  DenDF F value  Pr(>F)
## group_agent 3.4631  3.4631     1 3.1743  5.4574 0.09678 .
## ---
## Signif. codes:  0 '***' 0.001 '**' 0.01 '*' 0.05 '.' 0.1 ' ' 1
```
