# Supplementary material for: Evaluating generative AI for qualitative data extraction in community-based fisheries management literature
Source: Environ Evid. 2025 Jun 2;14:9. doi: 10.1186/s13750-025-00362-9 (PMC12128520; doi:10.1186/s13750-025-00362-9)
Supplement: Supplementary file 2 — Supplementary material 2. Statistical Analysis ($AF2_Statistical.pdf$AF2Statistical.pdf). Contains detailed statistical analysis including t-tests, linear mixed-effects models, and ANOVA results examining the effects of different AI implementations, question types, difficulty levels, and assessor types on extraction quality. [file 13750_2025_362_MOESM2_ESM.pdf]

Supplementary Materials for 'Evaluating Generative AI for Qualitative Data Extraction in Community-Based Fisheries Management Literature'

Supplement 1. Papers Included in the Review

1. Akimichi T. Indigenous resource management and sustainable development: Case studies from Papua New Guinea and Indonesia. Anthropological Science. 1995;103(4):321–7.

2. Almany GR, Hamilton RJ, Bode M, Matawai M, Potuku T, Saenz-Agudelo P, et al. Dispersal of Grouper Larvae Drives Local Resource Sharing in a Coral Reef Fishery. Current Biology. 2013 Apr;23(7):626–30.

3. Aswani S. Customary Sea Tenure in Oceania as a Case of Rights-based Fishery Management: Does it Work? Rev Fish Biol Fisheries. 2005 Aug;15(3):285–307.

4. Léopold M, Beckensteiner J, Kaltavara J, Raubani J, Caillon S. Community-based management of near-shore fisheries in Vanuatu: What works? Marine Policy. 2013 Nov;42:167–76.

5. Hamilton RJ, Hughes A, Brown CJ, Leve T, Kama W. Community-based management fails to halt declines of bumphead parrotfish and humphead wrasse in Roviana Lagoon, Solomon Islands. Coral Reefs. 2019 Jun;38(3):455–65.

6. Smallhorn-West PF, Stone K, Ceccarelli DM, Malimali S, Halafihi T, Bridge TCL, et al. Community management yields positive impacts for coastal fisheries resources and biodiversity conservation. CONSERVATION LETTERS. 2020 Nov;13(6)

7. Quimby B, Levine A. Adaptive capacity of marine comanagement: a comparative analysis of the influence of colonial legacies and integrated traditional governance on outcomes in the Pacific. Reg Environ Change. 2021 Mar;21(1):10.

8. Jupiter SD, Epstein G, Ban NC, Mangubhai S, Fox M, Cox M. A Social–Ecological Systems Approach to Assessing Conservation and Fisheries Outcomes in Fijian Locally Managed Marine Areas. Society & Natural Resources. 2017 Sep 2;30(9):1096–111.

9. King M, Fa'asili U. A network of small, community-owned Village Fish Reserves in Samoa.

10. Blythe J, Cohen P, Eriksson H, Cinner J, Boso D, Schwarz AM, et al. Strengthening post-hoc analysis of community-based fisheries management through the social-ecological systems framework. Marine Policy. 2017 Aug;82:50–8.

11. King M, Faasili U. Community-based management of subsistence fisheries in Samoa. Fisheries Management Eco. 1999 Apr;6(2):133–44.

12. Hoffmann TC. The Reimplementation of the Ra'ui: Coral Reef Management in Rarotonga, Cook Islands. Coastal Management. 2002 Oct;30(4):401–18.

13. Ison S, Hills J, Morris C, Stead SM. Sustainable financing of a national Marine Protected Area network in Fiji. Ocean & Coastal Management. 2018 Sep;163:352–63.

14. Smallhorn-West PF, Bridge TCL, Malimali S, Pressey RL, Jones GP. Predicting impact to assess the efficacy of community-based marine reserve design. CONSERVATION LETTERS. 2019 Jan;12(1)

15. Lauer M, Aswani S. Indigenous Knowledge and Long-term Ecological Change: Detection, Interpretation, and Responses to Changing Ecological Conditions in Pacific Island Communities. Environmental Management. 2010 May;45(5):985–97.

16. Albert S, Grinham A, Gibbes B, Tibbetts I, Udy J. Indicators of coral reef ecosystem recovery following reduction in logging and implementation of community-based management schemes in the Solomon Islands. Pac Conserv Biol. 2014;20(1):75.

17. Léopold M, Cakacaka A, Meo S, Sikolia J, Lecchini D. Evaluation of the effectiveness of three underwater reef fish monitoring methods in Fiji. Biodivers Conserv. 2009 Dec;18(13):3367–82.

18. Marine Ecology Progress Series 449:233. Mar Ecol Prog Ser. 2012.

19. Dumas P, Jimenez H, Léopold M, Petro G, Jimmy R. Effectiveness of village-based marine reserves on reef invertebrates in Emau, Vanuatu. Envir Conserv. 2010 Sep;37(3):364–72.

20. Doulman DJ. Community-based fishery management. Marine Policy. 1993 Mar;17(2):108–17.

21. Crean K. Centralised and community-based fisheries management strategies: case studies from two fisheries dependent archipelagos. Marine Policy. 1999 May;23(3):243–57.

22. Steenbergen DJ, Raubani J, Gereva S, Naviti W, Arthur C, Arudere A, et al. Tracing innovation pathways behind fisheries co-management in Vanuatu. Ambio. 2022 Dec;51(12):2359–75.

23. Johannes RE. The Renaissance of Community-Based Marine Resource Management in Oceania. Annu Rev Ecol Syst. 2002 Nov;33(1):317–40.

24. Williamson JE. Social attitudes towards marine resource management in two Fijian villages. 2006;7(2).

25. Hair C. Social and economic challenges to community-based sea cucumber mariculture development in New Ireland Province, Papua New Guinea. Marine Policy. 2020.

26. Fache E. Small-scale managed marine areas over time: Developments and challenges in a local Fijian reef fishery. Journal of Environmental Management. 2018.

27. Aswani S. One size does not fit all: Critical insights for effective community-based resource management in Melanesia. Marine Policy. 2017.

28. Rohe JR. Multiple Drivers of Local (Non-) Compliance in Community-Based Marine Resource Management: Case Studies from the South Pacific. Frontiers in Marine Science. 2017;4.

29. Robertson T, Greenhalgh S, Korovulavula I, Tikoibua T, Radikedike P, Stahlmann-Brown P. Locally managed marine areas: Implications for socio-economic impacts in Kadavu, Fiji. Marine Policy. 2020 Jul;117:103950.

30. Sulu RJ, Eriksson H, Schwarz AM, Andrew NL, Orirana G, Sukulu M, et al. Livelihoods and Fisheries Governance in a Contemporary Pacific Island Setting. PLOS ONE. 2015.

31. Hamilton RJ, Giningele M, Aswani S, Ecochard JL. Fishing in the dark-local knowledge, night spearfishing and spawning aggregations in the Western Solomon Islands. Biological Conservation. 2012 Jan;145(1):246–57.

32. Cohen PJ, Cinner JE, Foale S. Fishing dynamics associated with periodically harvested marine closures. Global Environmental Change. 2013 Dec;23(6):1702–13.

33. Ford AK. Evaluation of coral reef management effectiveness using conventional versus resilience-based metrics. Ecological Indicators. 2018.

Supplement 2. Extended Results

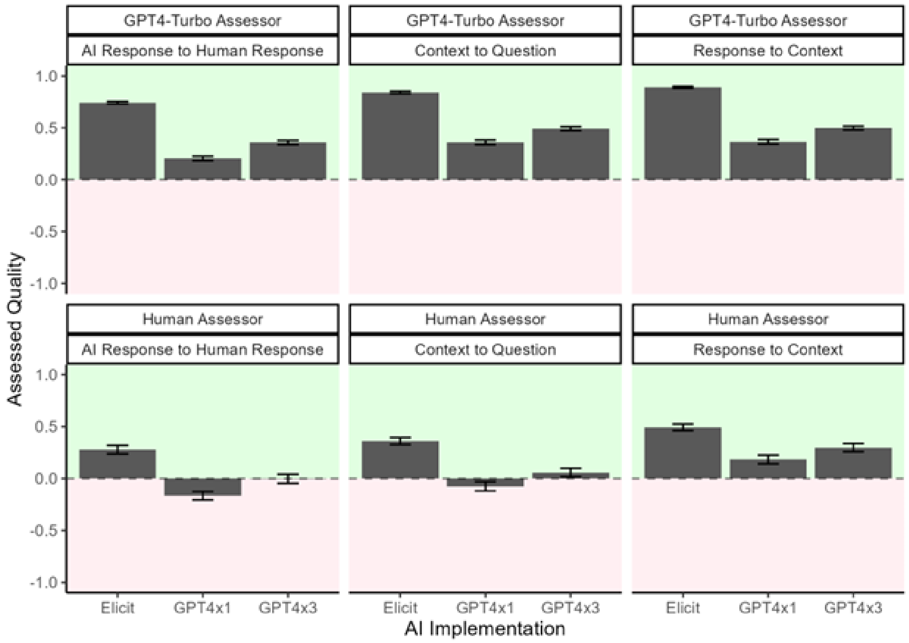

Figure 1: GPT-4 Quality Assessor. In addition to assessment by the team of human researchers. We had GPT-4 undertake the same assessment five times. (a) The first five times we provided the human response as the 'gold standard' and asked GPT-4 to assess the other response. These ratings are consistent in pattern to those of the human evaluators (Figure 3)

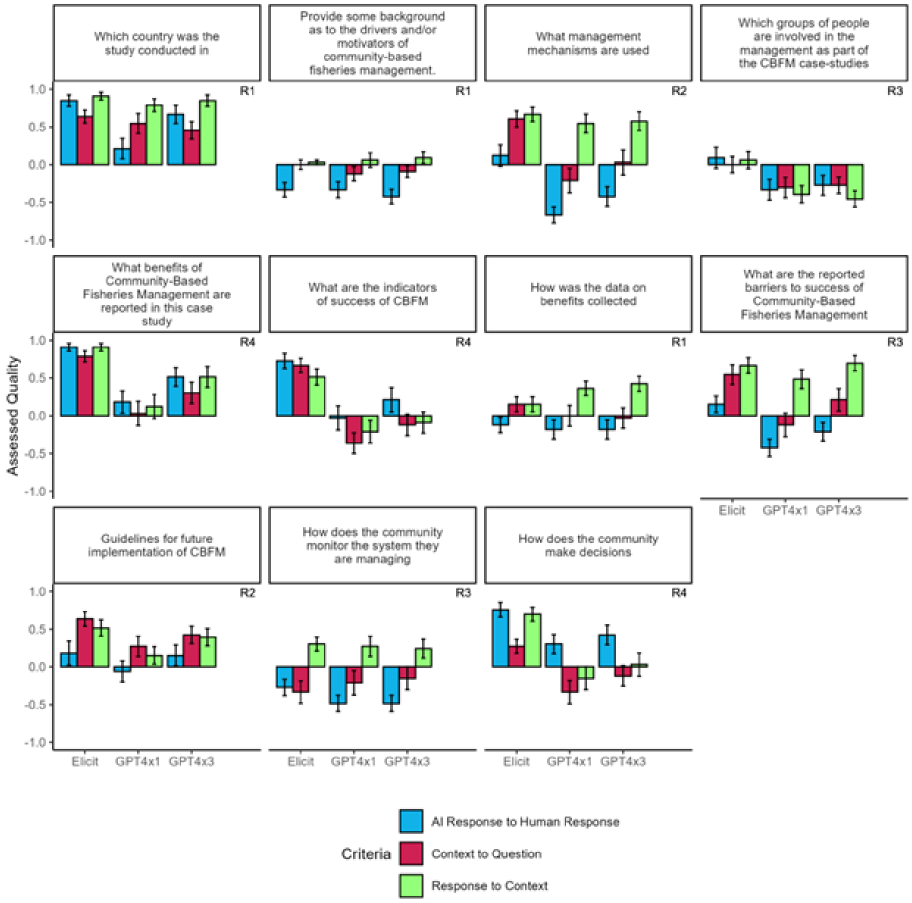

Figure 2: Mean Quality of AI responses for each question.
